# Supplementary material for: The Correlation of Frequency of Work-Related Disorders with Type of Work among Polish Employees
Source: Int J Environ Res Public Health. 2023 Jan 16;20(2):1624. doi: 10.3390/ijerph20021624 (PMC9861492; doi:10.3390/ijerph20021624)
Supplement: Supplementary file 1 [file ijerph-20-01624-s001.zip › File S1. The relationship between work and pain survey.pdf]

Informational post
Hello, We are fifth-year medical students at the Wroclaw Medical University conducting scientific study concerning correlation between type of work and and wrok-related disorders. We aim to explore the topic of correlation between type of work and and wrok-related disorders. We hope our study contributes to improvement of working conditions and workers' health. The survey involves some questions about your health problems, its intesity and frequency. The inquiry is fully-anonymous. Your responses will by statistically analysed, for scientific purpouse only.
By subbmiting this form you agree and consent to your personal data which you provide to the authors in this form beeing processed, collected, used, disclosed and retained by authors for scientific purpouse only.
If you are below the age of 18, the section above must be completed and acknowledged by your parent/guardian.
For more infromation on our Privacy Policy, please visit
https://www.umw.edu.pl/pl/kontakt/inspektor-ochrony-danych
Do not hesitate to contact us if you have any questions concerning the survey or study.
We appreciate your help.

The relationship between work and pain

We are fifth-year medical students at the Wroclaw Medical University, active in the Wroclaw Medical University Students' Scientific Society of Family Medicine. We aim to prove the association between work and prevalence of pain complaints. This survey is addressed to professionally active people, and it shouldn’t take longer than 5 minutes.

1. Sex
a. Woman
b. Man

2. Age range

options from 16 to 75 and above

3. Place of residence
a. Village
b. Town inhabited by 10.000-100.000 people
c. City more than 100.000 people

4. Education
a. Primary education
b. Lower secondary education
c. Secondary education
d. University education
e. University student

Work

5. What type of work do you perform?
    a. sedentary work
    b. work in a standing position
    c. physical work
    d. work in a forced position
    e. work that requires activity

Work-related pain

1. Do you experience work-related neck pain?
    a. Yes
    b. No
2. If you experience work-related neck pain, how often does it occur?
    a. not applicable
    b. less than once a year
    c. several times a year
    d. once a month
    e. once every 2 weeks
    f. once a week
    g. several times a week
3. If you experience work-related neck pain, what is the severity on a scale of 0 to 10, with 0 being no pain and 10 being unbearable pain?

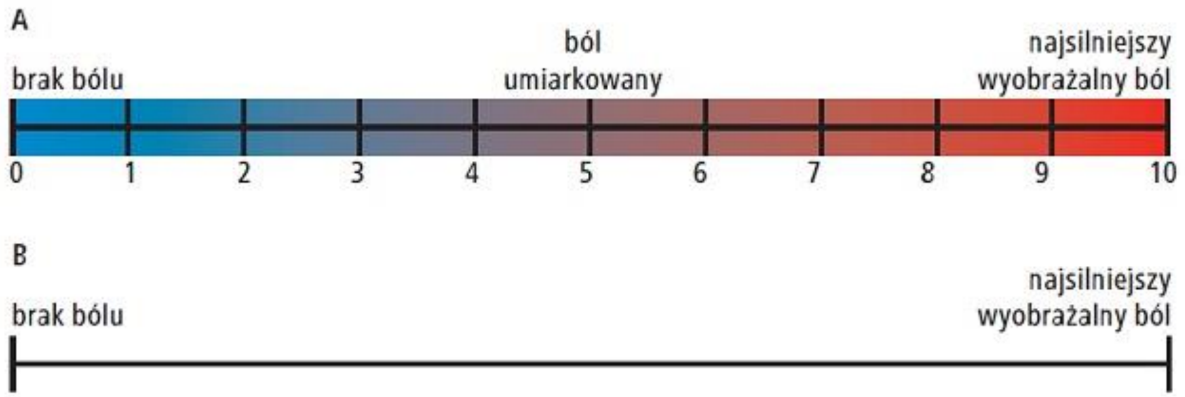

    a. Not applicable
    b. 0
    c. 1
    d. 2
    e. 3
    f. 4
    g. 5
    h. 6
    i. 7
    j. 8

    k. 9
    l. 10
4. Do you experience work-related thoracic (midback) pain?
    a. Yes
    b. No
5. If you experience work-related thoracic (midback) pain, how often does it occur?
    a. not applicable
    b. less than once a year
    c. several times a year
    d. once a month
    e. once every 2 weeks
    f. once a week
    g. several times a week
6. If you experience work-related thoracic (midback) pain, what is the severity on a scale of 0 to 10, with 0 being no pain and 10 being unbearable pain?

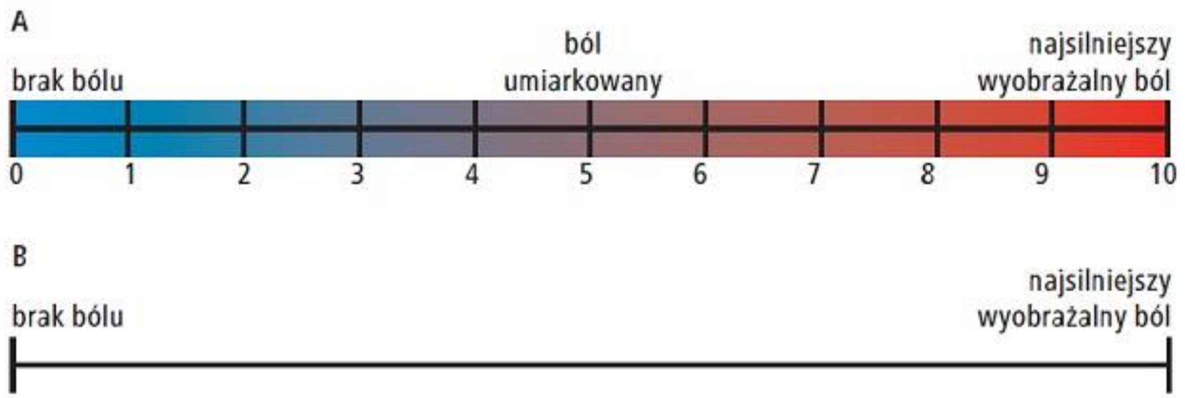

    a. Not applicable
    b. 0
    c. 1
    d. 2
    e. 3
    f. 4
    g. 5
    h. 6
    i. 7
    j. 8
    k. 9
    l. 10

7. Do you experience work-related lumbar (lowback) pain?
    a. Yes
    b. No

8. If you experience work-related lumbar (lowback) pain, how often does it occur?
    a. not applicable
    b. less than once a year
    c. several times a year
    d. once a month
    e. once every 2 weeks
    f. once a week
    g. several times a week

9. If you experience work-related lumbar (lowback) pain, what is the severity on a scale of 0 to 10, with 0 being no pain and 10 being unbearable pain?

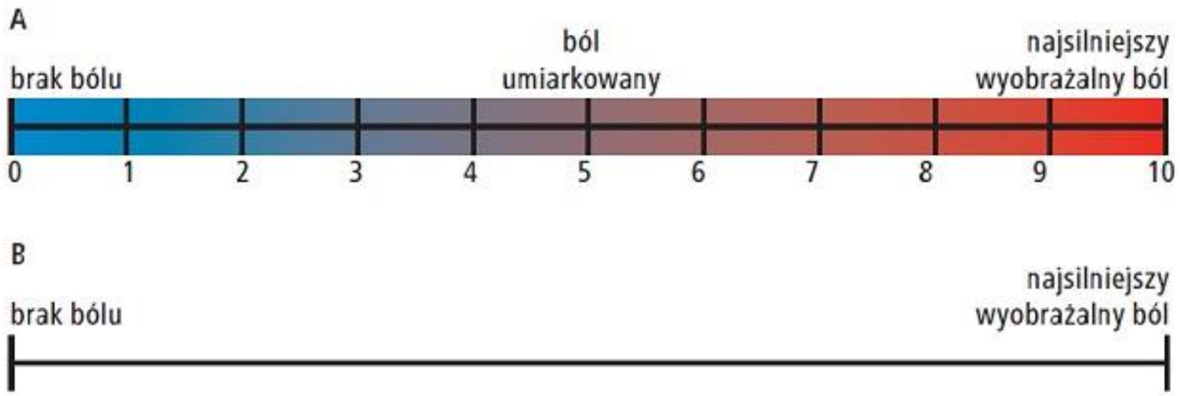

    a. Not applicable
    b. 0
    c. 1
    d. 2
    e. 3
    f. 4
    g. 5
    h. 6
    i. 7
    j. 8
    k. 9
    l. 10

10. Do you experience work-related shoulder pain?
    a. Yes
    b. No

11. If you experience work-related shoulder pain, how often does it occur?
    a. not applicable
    b. less than once a year

c. several times a year
d. once a month
e. once every 2 weeks
f. once a week
g. several times a week

12. If you experience work-related shoulder pain, what is the severity on a scale of 0 to 10, with 0 being no pain and 10 being unbearable pain?

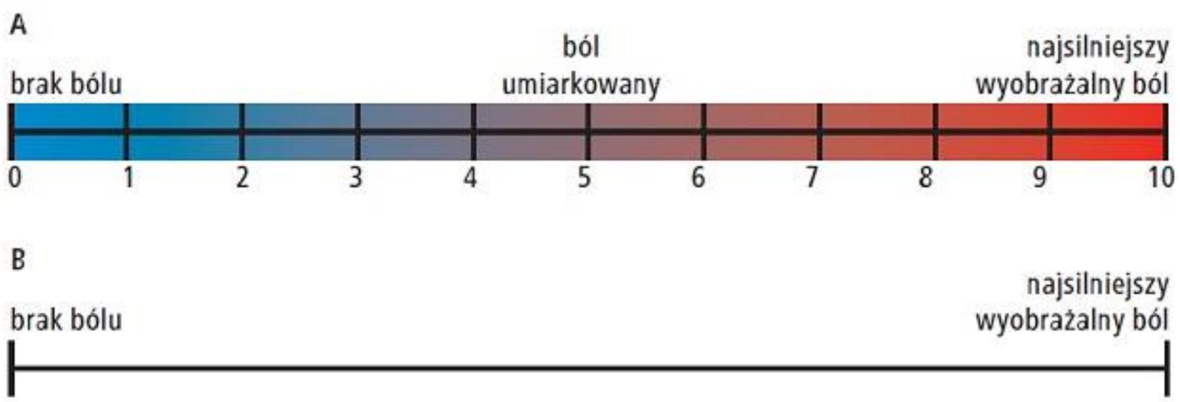

a. Not applicable
b. 0
c. 1
d. 2
e. 3
f. 4
g. 5
h. 6
i. 7
j. 8
k. 9
l. 10

13. Do you experience work-related elbow pain?
a. Yes
b. No

14. If you experience work-related elbow pain, how often does it occur?
a. not applicable
b. less than once a year
c. several times a year
d. once a month
e. once every 2 weeks

f. once a week
g. several times a week

15. If you experience work-related elbow pain, what is the severity on a scale of 0 to 10, with 0 being no pain and 10 being unbearable pain?

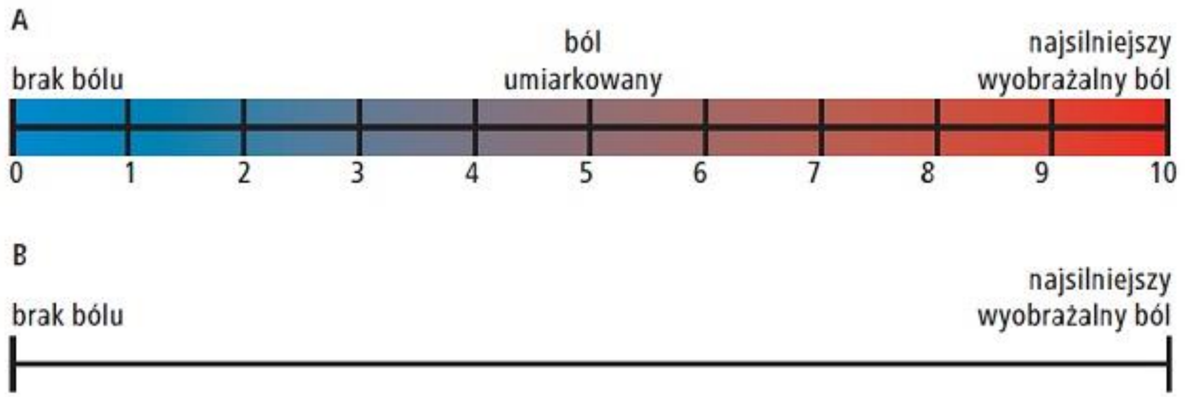

a. Not applicable
b. 0
c. 1
d. 2
e. 3
f. 4
g. 5
h. 6
i. 7
j. 8
k. 9
l. 10

16. Do you experience work-related hand joints pain?
a. Yes
b. No

17. If you experience work-related hand joints pain, how often does it occur?
a. not applicable
b. less than once a year
c. several times a year
d. once a month
e. once every 2 weeks
f. once a week
g. several times a week

18. If you experience work-related hand joints pain, what is the severity on a scale of 0 to 10, with 0 being no pain and 10 being unbearable pain?

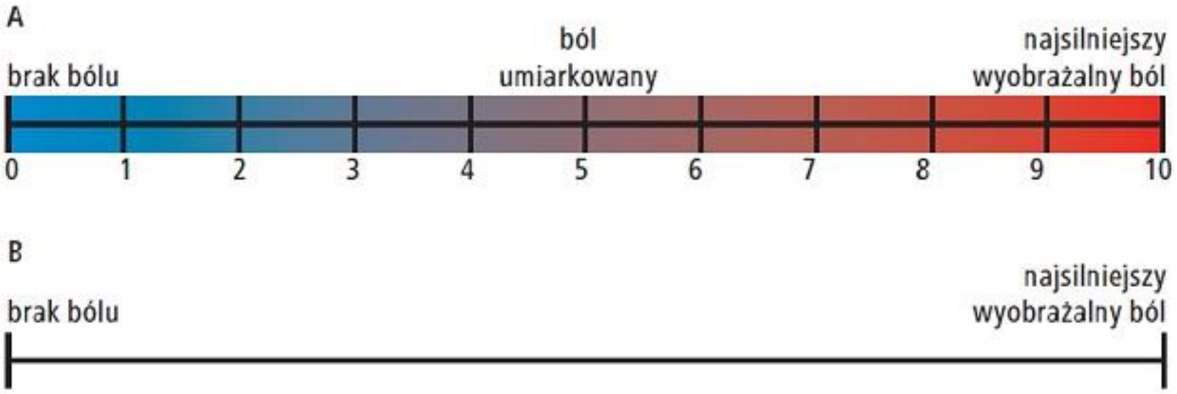

a. Not applicable
b. 0
c. 1
d. 2
e. 3
f. 4
g. 5
h. 6
i. 7
j. 8
k. 9
l. 10

19. Do you experience work-related hip pain?
    a. Yes
    b. No

20. If you experience work-related hip pain, how often does it occur?
    a. not applicable
    b. less than once a year
    c. several times a year
    d. once a month
    e. once every 2 weeks
    f. once a week
    g. several times a week

21. If you experience work-related hip pain, what is the severity on a scale of 0 to 10, with 0 being no pain and 10 being unbearable pain?

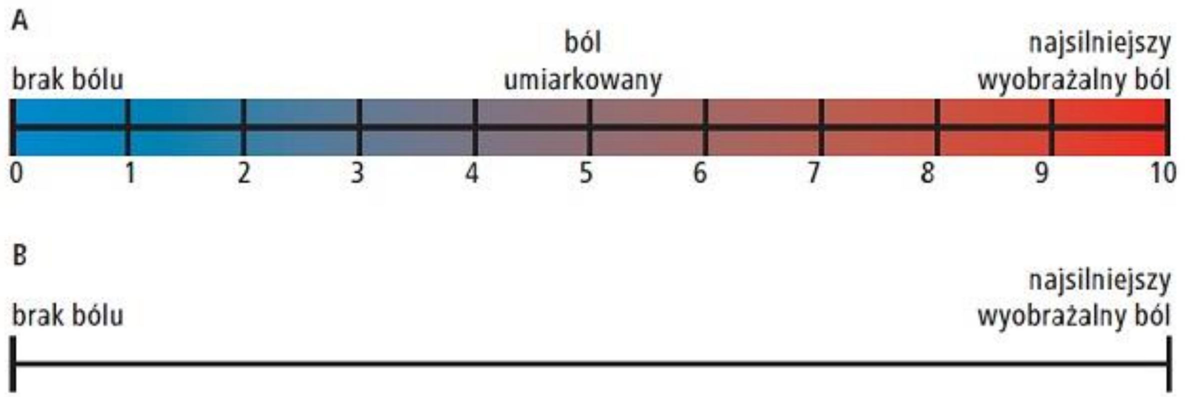

a. Not applicable
b. 0
c. 1
d. 2
e. 3
f. 4
g. 5
h. 6
i. 7
j. 8
k. 9
l. 10

22. Do you experience work-related knee pain?
    a. Yes
    b. No

23. If you experience work-related knee pain, how often does it occur?
    a. not applicable
    b. less than once a year
    c. several times a year
    d. once a month
    e. once every 2 weeks
    f. once a week
    g. several times a week

24. If you experience work-related knee pain, what is the severity on a scale of 0 to 10, with 0 being no pain and 10 being unbearable pain?

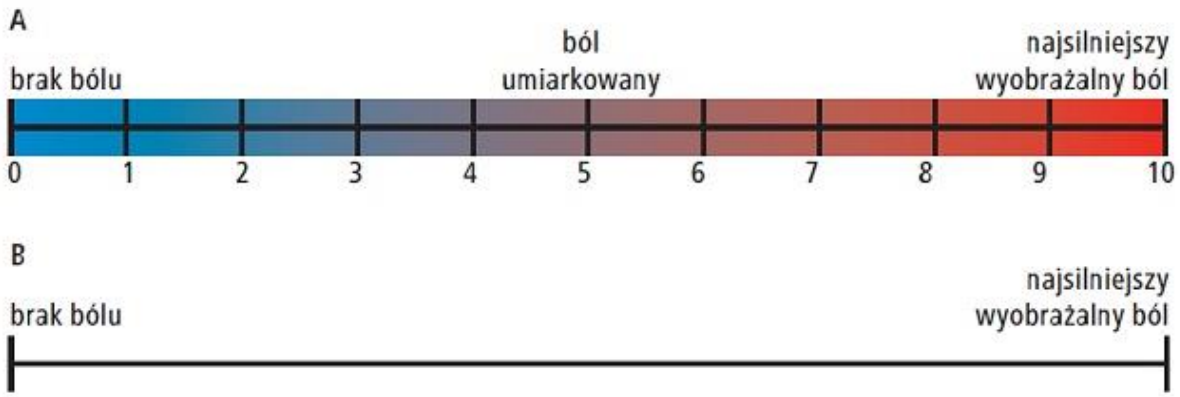

a. Not applicable
b. 0
c. 1
d. 2
e. 3
f. 4
g. 5
h. 6
i. 7
j. 8
k. 9
l. 10

25. Do you experience work-related ankle pain?
    a. Yes
    b. No

26. If you experience work-related ankle pain, how often does it occur?
    a. not applicable
    b. less than once a year
    c. several times a year
    d. once a month
    e. once every 2 weeks
    f. once a week
    g. several times a week

27. If you experience work-related ankle pain, what is the severity on a scale of 0 to 10, with 0 being no pain and 10 being unbearable pain?

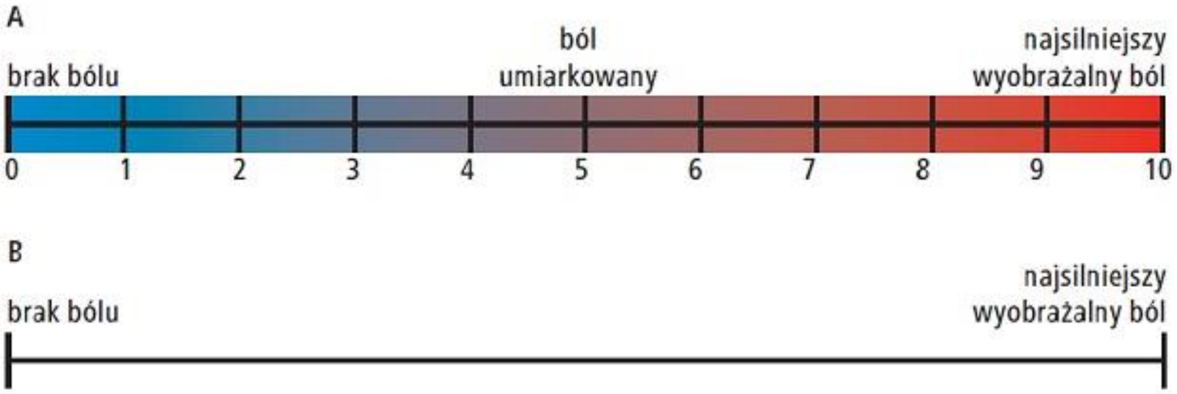

a. Not applicable
b. 0
c. 1
d. 2
e. 3
f. 4
g. 5
h. 6
i. 7
j. 8
k. 9
l. 10

28. Do you experience work-related lower extremity paresthesia (tingling, numbness, burning)?
    a. Yes
    b. No

29. If you experience work-related lower extremity parethesia (tingling, numbness, burning), how often does it occur?
    a. not applicable
    b. less than once a year
    c. several times a year
    d. once a month
    e. once every 2 weeks
    f. once a week
    g. several times a week

30. Do you experience work-related upper extremity paresthesia (tingling, numbness, burning) pain?
    a. Yes
    b. No

31. If you experience work-related upper extremity paresthesia(tingling, numbness, burning) how often does it occur?
    a. not applicable
    b. less than once a year
    c. several times a year
    d. once a month
    e. once every 2 weeks
    f. once a week
    g. several times a week

32. Do you experience work-related heaviness in the lower limbs?
    a. Yes
    b. No

33. If you experience work-related heaviness in the lower limbs, how often does it occur?
    a. not applicable
    b. less than once a year
    c. several times a year
    d. once a month
    e. once every 2 weeks
    f. once a week
    g. several times a week

34. Do you experience any of the listed lower extremity symptoms? (select all)

    a. telangiectasias (""spider veins"")

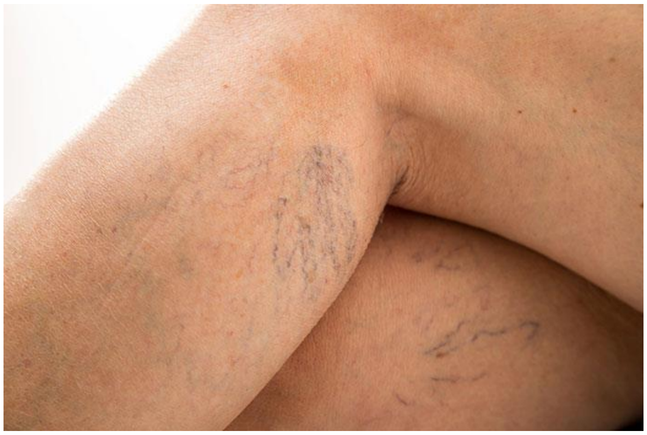

b. varicose veins

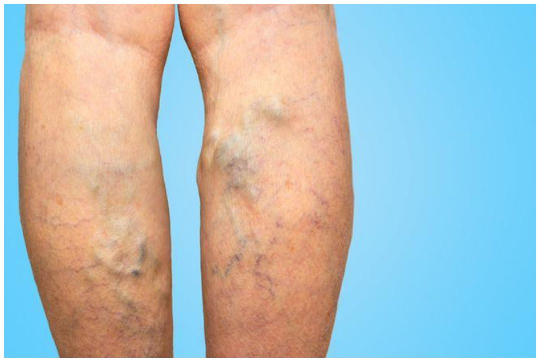

c. rusty-brown discoloration of the skin

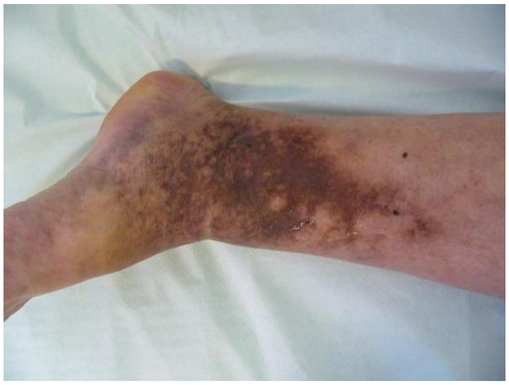

d. atrophie blanche

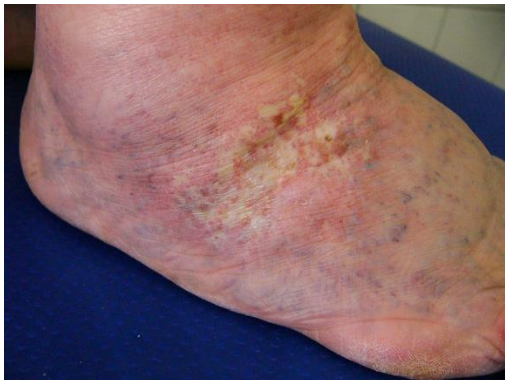

e. venous ulcers

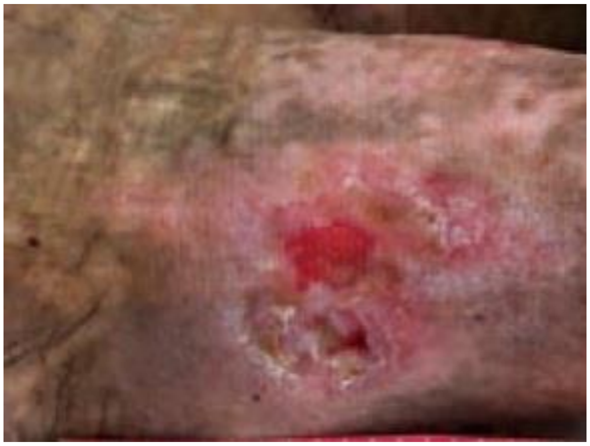

f. not applicable

35. Height in centimeters:

36. Weight in kilograms:

37. How much time per week are you spending on additional physical activity (walking, running, biking, gym, etc.)?
    a. I am not physically active
    b. 15 -60 minutes
    c. more than 1h, but less than 2h
    d. more than 2h, but less than 3h
    e. more than 3h, but less than 4h
    f. more than 4h, but less than 5h

38. Do you experience an increase in heart rate during additional physical activity?
    a. Yes
    b. No
    c. I don’t know

Sources:

1. https://adst.mp.pl/img/articles/strona-glowna/bol-635x269.jpg Accessed on 06 October 2022
2. https://mataba.pl/wp-content/uploads/2020/04/shutterstock_4704693621_easy-resize.com_.jpg Accessed on 06 October 2022

3. https://cdn.galleries.smcloud.net/t/galleries/gf-s4ZZ-8mYc-vwdg_zylaki-664x0-nocrop.jpg Accessed on 06 October 2022

4. https://marekciecierski.pl/wp-content/uploads/20040920-162834.jpg Accessed on 06 October 2022

5. https://marekciecierski.pl/wp-content/uploads/20061010-122337.jpg Accessed on 06 October 2022

6. https://adst.mp.pl/img/articles/zakrzepicazylna/rana-owrzodzenie.jpg Accessed on 06 October 2022
